# Automated Detection of Substance-Use Status and Related Information from Clinical Text

**DOI:** 10.3390/s22249609

**Published:** 2022-12-08

**Authors:** Raid Alzubi, Hadeel Alzoubi, Stamos Katsigiannis, Daune West, Naeem Ramzan

**Affiliations:** 1 Department of Computer Science, College of Computer Science and Information Technology, King Faisal University, Al-Ahsa 31982, Saudi Arabia; 2 Department of Computer Science, Durham University, Upper Mountjoy Campus, Stockton Road, Durham DH1 3LE, UK; 3 School of Computing, Engineering and Physical Sciences, University of the West of Scotland, High St., Paisley PA1 2BE, UK

**Keywords:** electronic health records, substance use, information extraction, natural language processing, machine learning, rule-based systems

## Abstract

This study aims to develop and evaluate an automated system for extracting information related to patient substance use (smoking, alcohol, and drugs) from unstructured clinical text (medical discharge records). The authors propose a four-stage system for the extraction of the substance-use status and related attributes (type, frequency, amount, quit-time, and period). The first stage uses a keyword search technique to detect sentences related to substance use and to exclude unrelated records. In the second stage, an extension of the NegEx negation detection algorithm is developed and employed for detecting the negated records. The third stage involves identifying the temporal status of the substance use by applying windowing and chunking methodologies. Finally, in the fourth stage, regular expressions, syntactic patterns, and keyword search techniques are used in order to extract the substance-use attributes. The proposed system achieves an F1-score of up to 0.99 for identifying substance-use-related records, 0.98 for detecting the negation status, and 0.94 for identifying temporal status. Moreover, F1-scores of up to 0.98, 0.98, 1.00, 0.92, and 0.98 are achieved for the extraction of the amount, frequency, type, quit-time, and period attributes, respectively. Natural Language Processing (NLP) and rule-based techniques are employed efficiently for extracting substance-use status and attributes, with the proposed system being able to detect substance-use status and attributes over both sentence-level and document-level data. Results show that the proposed system outperforms the compared state-of-the-art substance-use identification system on an unseen dataset, demonstrating its generalisability.

## 1. Introduction

Substance use (smoking, alcohol, and drug use) is a significant part of a patient’s history and can be used for clinical care and clinical research purposes. In order to improve patients’ health care, it is essential for clinicians to have a precise picture of each patient’s substance consumption. For instance, the value of the result of a common lab test (leukocyte differential count), which is used in the diagnosis of a variety of medical conditions, can cause confusion for clinicians if the patient’s status was not previously identified as a chronic alcoholic [1]. Moreover, substance use causes a wide range of medical conditions and can exacerbate existing ones. Various published clinical research works identify substance use as a risk factor that could lead to morbidity and mortality [2,3]. Smoking is associated with various diseases such as cardiovascular and gastrointestinal diseases, infections, and some types of cancer [4]. Consuming a large amount of alcohol has been shown to increase the risk of chronic pancreatitis, malnutrition, and cancer [5,6], whilst the long-term use of alcohol can seriously damage most organs and systems in the body [7]. Drug use effects include addiction, heart and/or lung disease, cancer, mental illness, hepatitis, and other serious health issues [8].

Substance use can be considered a disorder on its own if the frequent use of drugs and/or alcohol causes considerable clinical and/or functional impairment, such as health problems and disability [9]. According to the UK’s National Health Service (NHS), in England, there were 506,100 hospital admissions and 74,600 deaths attributable to smoking for 2019/20 [10]. Furthermore, according to the UK’s Office for National Statistics, in 2019, approximately 14.1% of the UK population aged 18 and above have smoked cigarettes [11]. For alcohol use, in 2017 in the UK, 7327 people died as a direct result of their drinking, but when every death in which alcohol was a factor is included, the figure is closer to 24,000 [12]. The misuse of alcohol is the biggest risk factor for death, ill-health, and disability among 15–49 year-olds in the UK and the fifth biggest risk factor across all ages [13]. In addition, alcohol-related illness is estimated to cost the NHS around GBP 3.5 billion annually in England alone [12]. For drug use, an estimated 1 in 11 adults aged 16 to 59 years had taken a drug in 2019 in the UK, equating to around 3.2 million people [14]. In the same year, there were 4393 registered deaths related to drug misuse in England and Wales [15]. Substance use can have a vast range of short- and long-term and direct and indirect effects. These effects vary based on the specific kind of substance used, how much is consumed, how it is consumed, and the overall health status of the user.

Electronic Health Records (EHRs) contain rich information about patients’ substance-use factors. This information can be either in a structured format or hidden within free text. EHRs are a source of valuable information about a vast range of diseases, health risk factors, and health outcomes [16]. This information enables researchers to conduct high-resolution interventional and observational clinical research [17,18,19]. However, most of the information is buried in unstructured narrative text [20]. Extracting information from clinical narrative text is a challenging and arduous task due to misspellings, unconventional abbreviations, and ungrammatical text [21]. In the last decade, Natural Language Processing (NLP) methods have received considerable attention as a way of analysing EHR narrative text and extracting the hidden information [16,18,22,23].

Recent developments in NLP techniques have demonstrated increasingly promising performance in the recognition and extraction of meaningful pieces of information from clinical narrative text [24,25]. NLP automates the processes required to access the vast embedded information in EHRs and to consolidate it into a coherent structure [26]. NLP techniques have been widely adopted in different fields, including medical applications such as cohort identification [27], genome-wide association studies [28], sentiment analysis [29], medical status extraction [30], text summarisation [31], and diagnosis code assignment [32]. A large number of tools and frameworks are currently available for clinical information extraction purposes, such as the *Clinical Text Analysis Knowledge Extraction System* (cTAKES) [33], *MetaMap* [34], and *Medical Language Extraction and Encoding* (MedLEE) [35]. Most frameworks employ dictionaries and rule-based techniques to identify clinical entities. The cTAKES system, which was developed by the Mayo Clinic, is one of the most popular open-source NLP systems in the literature. This system has been developed based on the *Unstructured Information Management Architecture* (UIMA) framework and the *OpenNLP* NLP toolkit; cTAKES is able to parse the clinical free text in order to identify the types of relevant clinical concepts in addition to qualifying elements such as “negated” or “non-negated” and “current” or “history”. The concept code is provided by mapping each class to a specific terminology domain, which is responsible for handling language variations.

A range of clinical NLP challenges have been organised in order to identify clinical entities from clinical text. The Centre of Informatics for Integrating Biology and the Bedside (i2b2) has organised many challenges. For example, in 2006, they presented a challenge for discovering patient smoking status [36]. In 2008, a challenge was conducted for recognising obesity from clinical text [37]. In 2009, the challenge focused on medication recognition [38]. In 2010, the challenge focused on recognising medical concept identification, classification, and relation extraction [39]. Other challenges have been shared by i2b2, such as the co-reference challenge [40], temporal relations challenge [29], and de-identification and heart disease risk factors challenge [41].

In this work, we propose a system to automatically detect smoking, alcohol, and drug-use status and the related information, such as amount, frequency, type, quit-time, and period, in medical records. Our system relies on several NLP techniques, such as chunking and keyword search, combined with rule-based methods to cope with complex clinical contexts. The developed system accurately extracts the information using four stages. In the first stage, the system excludes all records that do not contain any substance-use information using a keyword search method. In the second stage, an extension of the negation detection NegEx [42] algorithm is proposed. Temporal status (current or past) identification is conducted in the third stage, whereas in the last stage, the system extracts related substance-use attributes. Furthermore, the proposed system can work successfully at both the document-level and sentence-level. Our experimental evaluation on a dataset created and annotated for designing and testing our system, demonstrates the efficiency of the proposed system, achieving an F1-score of up to 0.99 for the detection of substance-use status and up to 0.98 for the detection of substance-use-related attributes. In addition, experimental evaluation on an “unseen” dataset demonstrates the generalisation ability of the proposed approach, achieving an F1-score of up to 0.97 for the detection of substance-use status and up to 0.99 or the detection of substance-use-related attributes. The contribution of our work can be summarised as follows: (a) We propose an automated substance-use detection system for clinical text and evaluate it for smoking, alcohol, and drug use. (b) We propose an extension of the NegEx negation detection algorithm that improves negation detection compared to the original algorithm. (c) We evaluate our proposed system on a dataset created and annotated for designing and testing our system as well as on an “unseen” publicly available dataset. (d) We evaluate our proposed system against deep learning approaches that use trainable or pre-trained word embeddings.

The rest of this paper is organised into four sections. The background information and related works are presented in Section 2. The proposed system is described in Section 3. Section 4 presents and discusses the obtained experimental results, whilst conclusions are drawn in Section 5.

## 2. Related Work

The use of EHRs has become critical in medicine, healthcare delivery, operations, and medical research [43]. However, the unstructured nature of the majority (80%) of information in EHRs makes it very difficult to process for secondary use [44]. The importance of being able to automatically process and extract information from unstructured clinical text has led to significant amounts of research work on the topic of text classification in the context of medical text and clinical notes. Available works rely both on traditional classification approaches and on more-recent neural-network-based classification and other advances in NLP [25]. Older works relied on classification approaches such as Support Vector Machines (SVMs) [45] and k-Nearest Neighbours (kNN) [46], while in recent years, deep-learning models such as Convolutional Neural Networks (CNNs) have attracted significant attention and have provided competitive results [47]. The use of pre-trained word embeddings such as word2vec [48] and GloVe [49], or attention-based models such as BERT [50,51], ClinicalBERT [52], EhrBERT [53], and BioBERT [54] have also recently been shown as viable alternatives [55].

Substance-use status identification has been widely investigated in the literature due to the associated health risks. Some systems detect substance use as a disease risk factor for heart disease [56,57,58,59], type 2 diabetes mellitus [60,61], and epilepsy [62], among others. Feller et al. [63] attempted to infer the presence and status of social and behavioural determinants of health (SBDH) information in patient records, including alcohol and substance use. In their work, clinical documents were represented as a bag-of-words using the term frequency–inverse document frequency (TF-IDF) representation. The L2-penalised logistic regression, SVMs, Random Forests, CaRT, and AdaBoost classifiers were then tested, achieving an F1-score of 91.3% for alcohol use detection and 92.5% for substance-use detection.

Aiming to identify smoking status, in 2006, i2b2 shared a dataset containing patient discharge summaries. The challenge set up was to classify patients’ records into five categories (“current smoker”, “past smoker”, “smoker”, “non-smoker” and “unknown”). Many systems were proposed to address this challenge, with Uzuner et al. [64] providing a summary of the reported results of these systems. Other later attempts were conducted on the same dataset to participate in the same challenge. Multiple classifications using Error Correcting Output Codes (ECOC) were used by Cohen [65] in an attempt to detect smoking status, achieving a micro-F1 score of 90%. His proposed system used a range of different techniques, including hot-spot identification, zero-vector filtering, inverse class frequency weighting, error-correcting output codes, and post-processing rules, with results showing that hot-spot identification had the largest positive effect. Wicentowski et al. [66] proposed a system to detect smoking status when smoking terms were present and when those terms were removed. Their system under-performed when the smoking terms were not available but performed similarly to expert human annotators. Heinze et al. [67] applied a medical coding expert system (LifeCode^®^) to the i2b2 smoking challenge. The authors concluded that smoking category temporal differentiation is a significant challenge. McCormick et al. [68] compared a supervised classifier using lexical features to one relying on semantic features generated by an unmodified version of MedLEE, a clinical NLP engine. Results showed that their supervised classifier trained with semantic MedLEE features was competitive with the top-performing smoking classifier in the i2b2 challenge, achieving an F1-score of 89%.

Sohn et al. [69] developed the Mayo smoking status-detection module by remodelling negation detection for non-smoker patients. Their system employed a rule-based model to classify document-level and patient-level smoking status. Their system works by employing a rule-based layer to detect the existence of smoking-related keywords. For any sentence with smoking information, a second layer is then used to detect any negation terms related to smoking terms using the NegEx algorithm. The SVM classifier is used to determine the sentence temporal status as past or current smoking. The portability of the Mayo module has been examined by Liu et al. [70] using Vanderbilt University Hospital’s EHR data; they concluded that modifications are necessary to improve the detection performance. Khor et al. [71] enhanced the Mayo smoking status-detection module to distinguish between current and past smokers. They retrained the SVM classifier by grouping keywords into lexically similar categories to improve efficiency and concluded that a keyword-grouping approach can increase the learning ability and enhance overall accuracy. Other approaches have compared the performance of systems that retrieve smoking status through different structured and unstructured sources [72,73]. SVMs were also used by Lix et al. [74] in combination with unigrams and mixed-grams to classify patient alcohol use from unstructured text in primary care EHRs. Results showed that a higher F1-score was achieved for classification of current drinkers using unigrams (89%) than mixed-grams (83%), but the difference for the unknown category was minimal (98% vs. 97%).

Wang et al. [75] developed an NLP system for detecting and extracting related information from substance-use sentences. The authors used the constituent parser and dependency parser along with rule-based methods to build a two-stage system. In the first stage, they detected three main subcategories of substance-use sentences (alcohol, drugs, and nicotine). In the second stage, they extracted attributes from the detected sentences, such as amount, frequency, status, method, and temporal information. They evaluated their system on two different datasets, MTSamples and notes from the University of Pittsburgh Medical Center (UPMC) de-identified clinical notes repository, achieving F1-scores of 89.8%, 84.6%, and 89.4% for alcohol-, drug-, and nicotine-use detection, respectively, as well as a maximum average F1-score of 96.6% for related attribute extraction. Yetisgen et al. [76] used a combination of machine learning and NLP approaches to identify sentences related to tobacco, alcohol, and drug use, as well as to their related attributes. Their system involved three steps: The first step detected the sentences that contained substance-use events. In the second step, they extracted seven entities to describe the events, and finally, in the third stage, they presented the extracted entities in a structured format. They evaluated their system on the MTSamples dataset, achieving F1-scores between 35.29% and 97.61%. One limitation of the aforementioned works is that they were designed for detecting sentence status rather than document status.

## 3. Methodology

### 3.1. Datasets and Annotation

To develop our system, we used a total of 1739 patient discharge summaries, acquired by merging two different previous i2b2 challenge datasets: (a) the smoking-status challenge dataset [36], which consists of 502 records covering outpatient, emergency room, and inpatient domains, and (b) the obesity-challenge dataset [37], which consists of 1237 records covering patients who were overweight or diabetic and had been hospitalised with obesity or diabetes. It must be noted that all patient records had been fully anonymised for the purpose of the challenges. The source data for both challenges originated from hospitals within the Partners Health-Care Research Patient Data Repository. The final merged dataset was divided into a training set of 1128 records (65%) and a test set of 611 records (35%).

The smoking challenge dataset was originally hand annotated by a team of pulmonologists. Every record was classified as one of five categories (unknown, non-smoker, smoker, current smoker, or past smoker) based on detailed criteria, and inter-annotator agreement was measured using Cohen’s kappa, resulting in a kappa value of 0.84. However, our proposed system deals with four categories (unknown, none, current, or past), where the smoker and current smoker categories have been merged; thus the dataset’s annotations were transformed accordingly. The annotation of our corpus was achieved in three phases: In the first phase, we followed the smoking challenge criteria to annotate the obesity challenge dataset for smoking status. In the second phase, all records from both datasets were annotated for alcohol- and drug-use statuses. In the third phase, we extracted five attributes (type, amount, frequency, quit-time, and period) for the records that positively held substance-use events.

**Figure 1:**
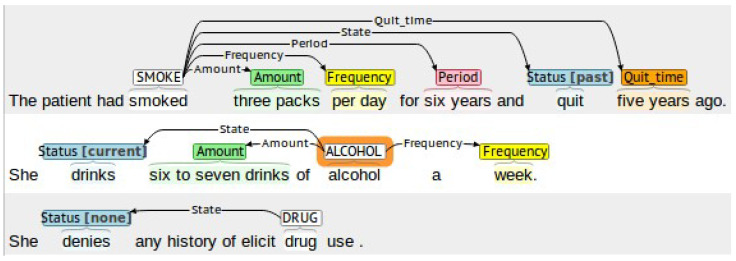
Example of using the BRAT annotation tool to annotate three sentences for smoking, alcohol, and drug use, respectively. Screenshot of the BRAT tool’s interface after annotating the three sentences. Using the tool, the parts of the sentence that refer to substance-use type, status, frequency, amount, period, and quit-time have been annotated.

To effectively annotate the dataset, we built annotation guidelines to annotate alcohol- and drug-use statuses and all attributes of substance use. The annotation guidelines were adapted from the annotation guidelines of the smoking challenge dataset [64]. Two clinicians performed a manual review of discharge summaries for 100 patients. The inter-rater agreement between clinicians was assessed by Cohen’s kappa, and the clinicians then met and resolved all conflicts. After that, a single annotator annotated the remaining patient records based on the revised annotation guidelines (Table 1) using the Brat Rapid Annotation Tool (BRAT) [77]. Figure 1 shows the annotation of a set of substance-use sentences using the BRAT, while Table 2 presents examples of substance-use attribute annotations.

A summary of the results of the aforementioned annotation procedure regarding the status of the discharge records is provided in Table 3. Out of the 1739 records, 800 (46%) were found to contain information regarding smoking, while the smoking status was unknown for 939 (54%) of the records. In addition, 628 (36.1%) records contained information regarding alcohol use, whereas 217 (12.5%) records contained information about drug use. Records related to any of the three substance-use types were those for which substance use could be established as “none”, “past”, or “current”, whereas unrelated records were those for which that the substance-use status was “unknown”. Out of the related records, 61.4%, 45%, and 20.7% were labelled as positive (current or past) records for smoking, alcohol, and drug use, respectively. Table 4 presents attribute annotation statistics for use of each substance in terms of the number of sentences that contained related information.

In addition to the two datasets that were merged in order to design and evaluate our system, we also employed the MTSamples [78] dataset in order to further evaluate our system, test its generalisability, and compare it to a state-of-the-art substance-use status-detection method on an “unseen” dataset. To this end, we used the annotated data provided by Yetisgen et al. [76].

### 3.2. Proposed Method

**Figure 2:**
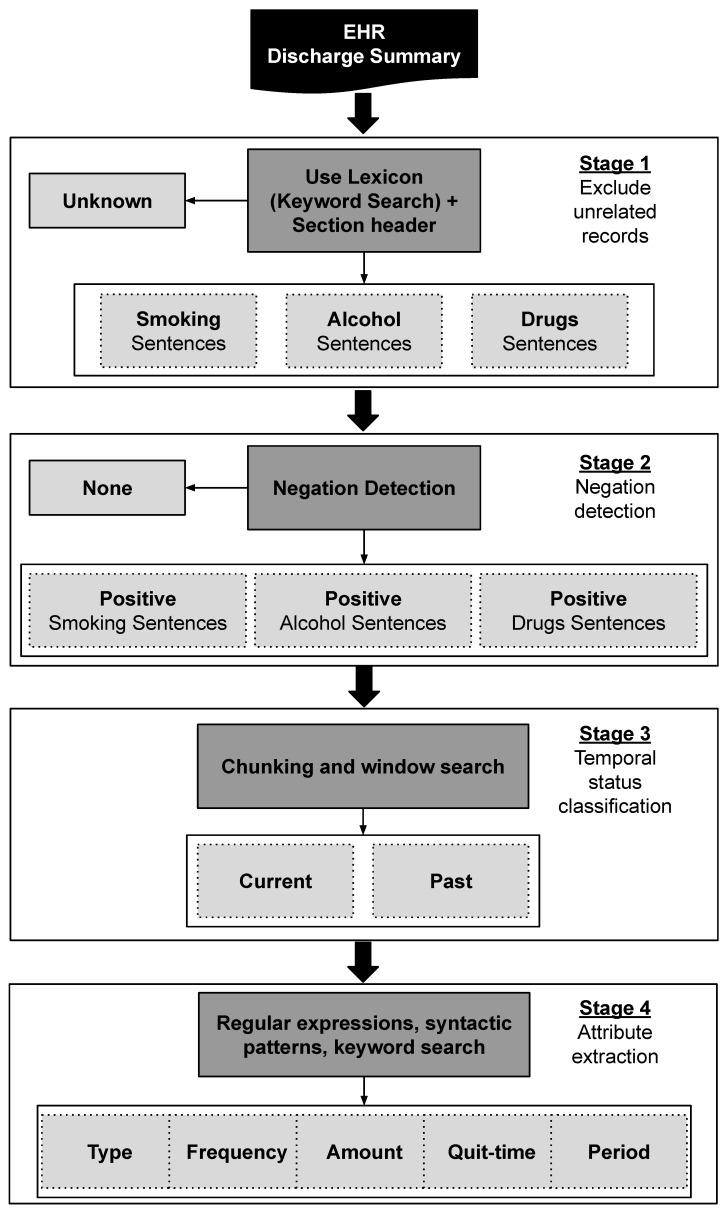
Flowchart of the proposed system for substance-use status and related attribute detection.

In this work, we aimed to develop an automated substance-use detection system for clinical text. The proposed system is able to detect the substance-use status and the related information from discharge summaries using a combination of NLP and classification rules. Our system consists of four stages, as depicted in Figure 2. In the first stage, the system classifies the records into two classes (“Known” or “Unknown”) for each substance use; all “Known” records are then processed further. The second stage detects the negation status and classifies the records into negative or positive. The third stage differentiates the temporal status of the positive records (past or current). In the final stage, the system extracts substance-use attributes (type, frequency, amount, quit-time, and period). Our system was developed using IBM’s Unstructured Information Management Applications (UIMA) engineering framework with text analysis components from Mayo’s cTAKES (e.g., tokeniser, sentence boundary detector, and shallow parser). Further details for each stage are discussed below.

#### 3.2.1. Stage 1—Exclusion of Unrelated Records

In this stage, the system classifies the records into known or unknown substance-use-related records by looking for specific keywords. For each type of substance use, the record is assigned an initial status. Records that are assigned a value of “Unknown” for the three substance-use classes (smoking, alcohol, and drug use) are discarded. Two lexicons have been built manually for alcohol and drug use from the training set, previously published studies [79], and expert domain knowledge (e.g., *alcohol, beer, ethanol, IVDU, cocaine, and heroin*). For the smoking lexicon, we used keywords from previously published work [69], and we also inserted new smoking-related keywords (e.g., *packs, ppd, snus, and weed*). A fourth lexicon was built that contains misleading concepts. These concepts include some of the keywords but with an inverse meaning in relation to substance use (e.g., *smoke inhalation, vaginal pack, drug allergies, and drug rash*).

An additional restriction was set in the case of alcohol and drugs. The proposed system uses section headers in the process of status detection. The patient records contain sections that may cause confusion in selecting the correct status. For example, the “ALLERGIES” section contains data about medication and drugs that cause allergic reactions in patients, but this information gives no clue for the existence of any substance use. Moreover, the detection system could deal with the keywords that exist in the “Follow up and Instructions” section that includes some instructions that the patient must follow after leaving the hospital. For example, in an instruction such as *“You should continue to take adequate food and drink”*, the keyword *“drink”* in this context does not mean the patient should or does consume alcohol. It must also be noted that the section headers were identified using a set of regular expressions.

Some keywords need to be treated carefully regardless of the section they appear in. For example, a list of different non-alcoholic drinks (e.g., *water, juice, and coffee*) deactivate the *“drink”* keyword. A shallow parser has been used to link the *“drink”* keyword with any adjacent word that appears in the non-alcoholic list. Another keyword that needs to be treated carefully is *“ppd”* because it could be the abbreviation of the *“Purified Protein Derivative (PPD)”* skin test or of *“packs per day”*. Our system needs to be concerned about the *“ppd”* keyword when it is connected to the patient’s amount of smoking. To solve this issue, the system uses a regular expression technique and considers *“ppd”* as a smoking keyword if it is preceded by a number, thus indicating the number of cigarette packs per day. Finally, the *“quit”* keyword can be ambiguous when it appears in a sentence without any substance-use keywords. Consequently, in this case, the system assigns the sentence containing *“quit”* to the preceding substance-use-related sentence.

#### 3.2.2. Stage 2—Negation Detection

In this stage, the system detects the negation status for all substance-use statements in the patient’s record, and a positive or negative status is assigned to every patient’s record. The records with positive status are transferred to the next stage, whereas the negative ones are discarded.

Negation is crucial for any clinical text analysis since it is widespread in the clinical narrative. For example, the medical importance of *“no smoking”* is quite different from that of *“smoking”*. NegEx [42] is one of the most widely used negation detection algorithms in the literature. In this work, we propose an extension of the NegEx algorithm, applied via the cTAKES negation annotator component, since the original NegEx failed to detect the negated substance records accurately in the proposed system. The negation annotator inspects the context of substance-use-named entities (e.g., smoking, alcohol, or drugs) to check if they have to be considered as negated. The best parameters for the NegEx annotator found throughout the training of our dataset were 9 for the maximum left scope and 2 for the maximum right scope of the substance keyword.

The extension proposed in this work aimed to address the limitations of NegEx by adding extra rules. The rules were proposed to handle the following cases:

- If semantic negation exists as words, the system fails to identify the status as negation, such as in the case of “nondrinker” and “non-drinker”. For example, *“She is a non-smoker and non-drinker”*. The “non-smoker” keyword was presented in Sohan [69]. We expanded the negation module by adding the new words to the negation component.
- NegEx fails to deal with negation when it appears as a sign (- or +), e.g., *-tob, +etho, or -IVDU*. Our negation module considers these signs as negation symbols while also taking into consideration the hyphen character “-”, e.g., *ex-smoker*.
- Solving the *“nor”* problem by expanding the negation window: For example, a sentence that contains *“nor smoke”* will be considered as negated, but the sentence *“She does not consume alcohol, nor does she smoke”* will not capture the negation for smoking since the smoking keyword is not directly connected with the context negation *nor*. We expanded the search after “nor” to connect it with the first appearance of a keyword.
- Solving the problem when the keyword of substance use is in a section header: The proposed system handles the case when the substance-use keyword is part of section header, e.g., *“Alcohol: None”, “Drug history: None”*. In the previous example, the status of alcohol use is none, even if the “alcohol” keyword exists in the section header.
- Solving complicated negation cases such as *“denies smoke, alcohol socially”*: This sentence indicates the patient is a non-smoker and current drinker. The original NegEx algorithm assigns negative status for smoking and alcohol use for the given patient. However, to address this obstacle, the system was extended to include words that override negation, i.e., *socially, rare, or occasional*.
- Terms used to override negation: For example, *“he has not drunk alcohol since 1984”*. In the previous sentence, the patient is a past drinker, while NegEx considers the patient as a non-drinker. To address this issue, the system was extended to include words that override negation, i.e., *since, for, last, or quit*.
- The case when the sentence includes more than one keyword for the same substance use, causing a conflict when assigning a negation status. For example, *“he is a former smoker, he denies any use of tobacco”*. This sentence indicates that the patient is a past smoker. NegEx will give the first keyword *“smoker”* positive status and the second keyword *“tobacco”* negative status. To handle this issue, the proposed system uses the ’OR’ logical operator so that positive status will be considered for the whole sentence whenever it exists. Moreover, since our system detects the status of the documents, the probability of having more than one keyword for the same substance-use-related sentence is higher, which makes the use of the ’OR’ operator in the proposed system essential.

After applying negation detection, the documents are given a certain status (positive or negative). The documents with positive status are then transferred to the next stage.

#### 3.2.3. Stage 3—Temporal-Status Classification

This stage contains documents that have a positive appearance of substance-use keywords. These documents will be classified into “Current” or “Past” for each substance use. At the beginning, positive sentences are considered to be current substance users unless clear past evidence is presented. There are some substance-use treatment types that indicate that the patient is a current substance user, such as *“nicotine patch”, “smoking cessation”, and “ethanol abstinence”*. Other keywords can give a clue about the current status, such as *“to stop” and “to quit”*. The document is assigned a past substance-use status if one of the past terms such as *“ex-smoker” and “ex-drinker”* appears. Window and chunking methodologies were used to link substance-use keywords with past terms. A list of the past terms and search methodologies used is provided in Table 5.

In cases where the sentence includes keywords of various types of substance use, the past status is assigned to the closest one. Drug-use temporal status identification is more complicated in cases where the patient could be a past user of one drug and a current user of others. In this case, the past status will be linked to the particular type of drug(s). The final judgment for drug-use status is “Current” if the patient currently uses any kind of drug(s).

#### 3.2.4. Stage 4—Attribute Extraction

In this stage, the proposed system extracts the related attributes for smoking, alcohol, and drug use. The extracted attributes are: amount, type, frequency, period, and quit-time. These attributes are extracted from records with current and/or past status for every type of substance use. Different rules have been implemented to extract these attributes as follows:

- *Amount* is the quantity of smoking, alcohol, or drug consumed by the patient. To extract the amount attribute, three levels of scanning are conducted. Firstly, the system searches for patterns using a regular expression to detect instances such as *“1 to 2 packs”, “3–4 drinks”*. A second scan is applied to deal with cases for which the amount is not a numeric value, such as *“five packs”, “two to three beers”*. In this scan, a shallow parser is used to find the longest chunk that contains an amount value. Then, the system searches for syntactic patterns for amount values, e.g., *“((CD to) CD packs)”, “(CD drinks)”*. If the system fails to detect amount values, a third scan searches for lexical words such as *( “heavy”, “minimal”, “significant”)* that are occasionally used to describe the amount value in the place of numbers.
- *Type* is the kind of smoking, alcohol, or drug(s) consumed by the patient. For the type attribute, we used a lexicon to identify the substance-use type. The lexicon terms were collected from previously published work [79] and expanded by adding other terms selected manually from the training set with the help of experts. The lexicon is divided into three parts, one for each type of substance use. Example terms are *cigarette, pipe* for smoking, *wine, vodka* for alcohol, and *crack, cocaine* for drugs.
- *Frequency* is how often smoking, alcohol, or drugs are used by the patient. The frequency attribute commonly follows the amount value and comes in the same chunk. To extract the frequency value, a regular expression and syntactic pattern such as ((amount value) | times) (per | a | / | every | -) (day | week | year | month) are used. If these patterns are not found, a second scan is conducted to find lexicon words such as *“occ”, “nightly”, “infrequently”*.
- *Quit-time* and *Period*: *Quit-time* is the date that the patient stopped substance use, and *period* is the duration of substance use. To extract quit-time and period attributes, the system searches for a syntactic pattern such as ((quit | stop | discontinued | until ) (CD)), (for | times (CD)). Moreover, the system searches for some regular expression patterns to solve cases such as “× 20 yrs” for period and *“5 years ago”* for quit-time. When a sentence contains keywords for various types of substances use, the quit-time and period values will be assigned to the adjacent substance use type or the nearest prior type listed.

### 3.3. Novelty and Validation

The proposed system is generalisable, having been developed and trained over the i2b2 dataset and tested using an unseen part of the i2b2 dataset (test set), as well as the completely unseen and independent MTSamples dataset. As shown in the next section, it is able to perform better than other state-of-the-art systems. The proposed system is enhanced in the sense that it: (1) extends the NegEx algorithm; (2) expands the keyword lexicon by investigating the training set, previous works, and domain knowledge experts; (3) applies special rules for misleading keywords; (4) expands the search to all text sections and links the keywords with their own section header; and (5) employs a shallow parser in extracting substance-use status and attributes.

## 4. Results and Discussion

A thorough experimental evaluation was conducted in order to examine the performance of the proposed system on the tasks of detecting unknown, none, past, or current substance use (smoking, alcohol, and drugs) from discharge records as well as on the task of detecting substance-use attributes (amount, type, frequency, quit-time, and period). To ensure a fair experimental evaluation and to avoid overfitting, the dataset was divided into training (65%) and test (35%) sets. To this end, 1128 records were used to design and fine-tune our system, and 611 records were used to test the system’s performance. For smoking, the number of related records was 503 records for training and 297 records for testing. For alcohol, there were 389 records for training and 238 testing records. Finally, for drugs, there were 128 records for training and 89 for testing. The performance of our system was measured using the following information retrieval metrics: sensitivity (Sen), precision (Pre), and F1-score (F1).

### 4.1. System Performance Evaluation

The performance of our proposed system on the test set is reported in s Table 6 and Table 7. Our system was able to accurately detect related substance-use records (Stage 1) with an F1-score between 0.9785 and 0.9974. For smoking and alcohol use, the proposed system detected all related records correctly with a sensitivity of 1.0, while sensitivity reached 0.9785 for drug use detection. Precision exceeded 0.97 for all substance-use detection types, resulting in very few unrelated records being retrieved.

In order to identify the negated records (Stage 2), we first tested using the cTAKES NegEx method. However, NegEx failed to identify the negated records. For instance, when identifying negated smoking records, 11 records were identified out of 113. Results were similar for identifying negated alcohol records, where only 9 out of 135 were detected correctly. In addition, none of the negated drug records were identified. We then tested our proposed negation-detection method. As shown in Table 6, our negation-detection module (Stage 2) was successful in detecting negation, achieving an F1-score between 0.9710 and 0.9821. Sensitivity reached 0.9735 for detecting negation for smoking, 0.9630 for alcohol use, and 0.9571 for drug use. Furthermore, our proposed extended negation-detection module reduced false positives significantly, achieving a precision value of more than 0.98.

Temporal status classification into current or past (Stage 3) was the most challenging stage for our system. The proposed system was able to detect the smoking temporal status with an F1-score of 0.9463 for “Past” and 0.9294 for “Current” statuses, thus achieving an average F1-score of 0.9379. For the detection of alcohol-use temporal status, the average F1-score was 0.8731, with an F1-score of 0.80 for “Past” and 0.9461 for “Current” statuses. Finally, for the detection of the drug-use temporal status, the average F1-score was 0.8286, with an F1-score of 0.8571 for “Past” and 0.80 for “Current” statuses. From these results, it is evident that the proposed system is more efficient in detecting the temporal status for smoking compared to alcohol and drug use.

The performance of the proposed system for the extraction of substance-use attributes (Stage 4) is presented in Table 7. Attributes related to smoking were accurately identified, with an F1-score between 0.9244 and 1. For alcohol use, the proposed system successfully identified the amount, type, and frequency attributes with an F1-score between 0.9333 and 0.9831, while performance suffered for the quit-time attribute, reaching an F1-score of 0.80. This decrease in performance can be attributed to the various syntactic patterns appearing in alcohol-related text. Finally, the proposed system was successful in identifying the amount, type, and period attributes for drug use, achieving an F1-score between 0.8780 and 1, with performance dropping for the quit-time attribute, reaching an F1-score of 0.75. It must also be noted that the achieved precision was very high in most cases, indicating that our system returned a very small number of incorrect results.

### 4.2. Performance Comparison to Deep Learning Approaches

To further evaluate the suitability of the proposed system, its performance was compared to that of six deep learning approaches for the task of substance-use status classification. To this end, the task was modelled as a four-class classification problem with the classes being “Unknown”, “None”, “Past”, and “Current”. The following six models were then trained for each one of the three substances in the dataset following the approaches of [80,81,82]: (i) a Multilayer Perceptron (MLP) model using a trainable embedding layer, (ii) a Bidirectional Long Short-Term Memory (Bi-LSTM) [83] model using a trainable embedding layer, (iii) an MLP model using the GloVe-50 [49] pre-trained word embeddings, (iv) a Bi-LSTM model using the GloVe-50 pre-trained word embeddings, (v) the pre-trained Bidirectional Encoder Representations from Transformers (BERT) [50] base uncased model, and (vi) a fine-tuned BERT base uncased model. The trainable embedding layer used in the first two models had 128 dimensions. In addition, a sequence length of 512 tokens was used for BERT, as it was the maximum supported by the BERT base uncased model, whereas the sequence length for the MLP and Bi-LSTM models was set to 2500 tokens. Cross-entropy loss and the Adam optimiser were used for training the models using the Keras API, with the loss function being weighted according to the class ratios in order to account for the class imbalance. It must be noted that in order to achieve a fair comparison to the proposed approach, no additional preprocessing was applied to the text. Each text was first tokenised, and the list of tokens was used as an input to the models. Results for each substance and for each class for all the examined deep learning models are presented in Table 8.

From Table 8, it is evident that the examined deep learning approaches performed significantly worse that the proposed method. For detecting “Unknown” use status, the fine-tuned BERT model performed the best for smoking and alcohol use, with F1-scores of 0.7611 and 0.80, respectively, and the MLP with trainable embedding performed the best for drug use, with an F1-score of 0.9442, compared to 0.9969, 0.9974, and 0.9785, respectively, for the proposed method. For the detection of negated records (“None”), the fine-tuned BERT model performed the best for smoking, with an F1-score of 0.4768, and the MLP with trainable embedding performed the best for alcohol and drug use, with F1-scores of 0.3866 and 0.2424, respectively, compared to 0.9821, 0.9738, and 0.9710, respectively, for the proposed method. For the detection of “Past” use status, the MLP with trainable embedding performed the best for smoking and drug use, with F1-scores of 0.2264 and 0.50, respectively, and the MLP with GloVe-50 embeddings performed the best for alcohol use, with an F1-score of 0.0769, compared to 0.9463, 0.8571, and 0.80, respectively, for the proposed method. It must be noted that for the detection of “Past” status for drug use, the Bi-LSTM with trainable embeddings and the MLP with GloVe-50 embeddings also achieved an F1-score of 0.50. Finally, for the detection of “Current” use status, the BERT model performed the best for smoking, with an F1-score of 0.2072, the fine-tuned BERT performed the best for alcohol use, with an F1-score of 0.2873, and the MLP with GloVe-50 or trainable embeddings performed the best for drug use, with an F1-score of 0.1818, compared to 0.9294, 0.9461, and 0.80, respectively, for the proposed method. It is evident that, apart from the detection of records with “Unknown” substance-use status, the examined deep learning approaches struggled to distinguish between the other classes, something that can be partially attributed to the lack of sufficient training samples (as shown in Table 8) combined with the high dimensional feature space due to the length of the medical discharge records.

### 4.3. Evaluation on an Unseen Dataset

In order to demonstrate our system’s generalisability and superiority, we tested our system using the MTSamples dataset [78] that has also been used by Yetisgen et al. [76]. Yetisgen et al. employed machine learning for the detection of the substance-use status, as previously mentioned in the related works (Section 2). Table 9 provides a comparison between the results of our proposed system on the MTSamples dataset and the results published by Yetisgen et al. for the same dataset. Since the compared work detects the status at the sentence level, we also configured our proposed system to detect the status from sentences instead of records in order to conduct a fair comparison. It is evident from Table 9 that the proposed system outperforms Yetisgen et al.’s approach, achieving a higher F1-score in all cases except for alcohol use with a status of “Current” and drug use with a status of “None”. It must also be noted that our system led to significant improvements in F1-scores in most cases compared to Yetisgen et al.’s and to more stable performance across the three different statuses for each substance.

In addition to detecting the substance-use status from the MTSamples dataset, we also extracted the related substance-use attributes. Results are provided in Table 10 and are consistent with the results achieved for the dataset used to create and evaluate our system (Table 7). The generalisability of systems and models across different datasets is one of the greatest challenges in this field. The results of our proposed system on an unseen independent dataset demonstrate its generalisation ability, indicating its potential for real-world use.

### 4.4. Error Analysis

By analysing the performance of each stage of our system, we identified the sources of errors as follows:

In the first stage, the proposed system fails to exclude the records when the substance-use keywords that appear in the text belong to one of the patient’s family members, such as father, mother, or wife. The cTAKES assertion module detects the main subject of the statement. However, we avoid using this module to identify the family member in our system because it dramatically increases the false-positive rate. Another source of error is misspelled keywords. For example, when identifying drug-related records, the system fails to include records that contain misspelled keywords such as “*barbituate abuse*”. Moreover, while the system sets extra restrictions over the patient record sections in detecting drug-related keywords, these keywords could appear in one of the restricted sections. For example, the existence of *“remote drug abuse”* could appear in the section *“HISTORY OF PRESENT ILLNESS”*.

In the remaining stages, the main error source that increases false positives and false negatives is the case when sentences include keywords of different substance use types. For example, in negation detection, the system fails to detect the following negation correctly: *“Smoking, no alcohol”*. While the previous sentence means that the patient is a smoker and does not drink alcohol, the system assigns negative status for both smoking and alcohol keywords. In some cases where the negation word is too far from the substance-use-related keywords, the system did not detect the negation. For example in the sentence *“Abuse of drugs or alcohol were denied”*, the word *“drugs”* is too far from *“denied”* to be negated, as our system uses a two-word window after and nine-word window before the substance-use keyword to detect negation. However, expanding the window after the keyword is likely to increase the false-positive rate.

Finally, some error sources come from patterns that did not appear in the training set. Since drug abuse is not as widespread as smoking and alcohol use, it is normal to have fewer samples in the dataset. In our case of 1739 records, only 45 were identified as patient drug-use records. Moreover, out of these 45 records, the frequency of drug use was mentioned only in 4 records. Consequently, our system was not able to detect the drug-use frequency value due to the absence of any cases in the testing set. The same situation was also faced when identifying alcohol-use period values.

### 4.5. Further Discussion

From Table 8, it is evident that the examined deep learning models underperfomed significantly compared to the proposed rule-based system. We hypothesise that this is a result of the combination of an insufficient number of samples, especially for some pairs of substance and class, and the large size of the patient discharge summaries. The maximum length of the discharge summaries in the dataset was 3821 words and the median length was 948 words. For the MLP and Bi-LSTM models, the number of words led to a significantly large feature space, especially after their conversion to their respective word embeddings. The large feature space combined with the small number of samples for some pairs of substance and class plus the overall class imbalance hindered the performance of the MLP and Bi-LSTM models and led to overfitting. In addition, despite BERT not being affected by the size of the word representation, the limitation of BERT’s base uncased pre-trained model to inputs of a maximum of 512 tokens resulted in the model ignoring a significant amount of text in the case of larger discharge summaries, resulting in decreased performance. Furthermore, the use of pre-trained word embeddings, such as GloVe, may lead to discarding some valuable words, such as abbreviations, names of substances, and medical terms, as these words may not exist in the corpus used for training the embeddings. Indeed, results in this work showed that the two models with trainable embeddings performed better that the same models using the GloVe-50 embeddings.

## 5. Conclusions

In this work, we built a system to automatically detect substance use (smoking, alcohol, and drug use) and to extract related attributes from clinical text, more specifically, from patient discharge records. A suitable dataset was created and annotated and was finally divided into a training set that was used for designing and fine-tuning the proposed system and a test set that was only used for the final performance evaluation. The proposed system was built using NLP and rule-based techniques and consists of four stages: In the first stage, the system excludes unrelated records using a keyword search technique, achieving an F1-score of up to 0.99. In the second stage, an extension of the NegEx negation detection algorithm detects the negation status of patient records, achieving an F1-score of up to 0.98. In the third stage, the system extracts the temporal status of the substance use and classifies the records into current or past status, achieving a maximum F1-score of 0.94. Finally, the substance-use-related attributes (amount, frequency, type, quit-time, and period) were extracted from each patient record using rule-based techniques, achieving satisfactory performance.

The experimental evaluation showed that the proposed system is able to successfully detect the substance-use status and related attributes from clinical notes at both document-and sentence-level. Furthermore, in order to demonstrate its generalisability, the proposed system was also evaluated on the independent, unseen MTSamples dataset, and its performance was compared with a state-of-the-art approach. Results showed that the proposed system outperformed the compared approach on an unseen dataset, thus demonstrating its generalisation ability. To conclude, the results suggest that using NLP and rule-based techniques is an efficient way of extracting embedded information from clinical text.

For future work, we hypothesise that working with a larger dataset could potentially improve the ability to generate more rules and thus increase the system’s overall performance. Furthermore, apart from the rule-based approach, we plan to work on deep learning approaches for the task at hand. The mediocre performance achieved by the examined deep learning approaches in this work demonstrated various shortcomings that must be addressed. The large size of the clinical discharge records, in combination with the limited number of samples for some pairs of substance and use status, hindered the ability of state-of-the-art deep learning models to perform efficiently. To this end, we plan to increase the size of the available training datasets by gathering and curating additional clinical discharge records; we also plan to develop deep learning approaches tailored to the examined task. Finally, we plan to examine whether a hybrid approach that combines rules-based and deep learning approaches for different stages of the processing pipeline may be more suited for the examined task by training separate deep learning models for each stage of the pipeline and combining them with rule-based methods. In addition, the issue of the large text length could be potentially addressed by training the models at sentence-level, thus significantly reducing the number of input tokens, and then using a rule-based strategy to combine the statuses of all sentences in a text.

## Figures and Tables

**Table 1:** Substance-use status annotation guidelines.

Category	Annotation Guideline
Current	A “Current” substance user is a patient whose discharge summary asserts explicitly that the patient had used the substance within the past year. The assertion “Current [substance] user” in the text without any temporal qualifications means that the patient is categorised as a “Current” substance user unless the text states that the patient stopped using the substance more than a year ago.
Past	A “Past” substance user is a patient whose discharge summary asserts explicitly that the patient was a substance user one year or longer ago but who has not used the substance for at least one year. The assertion “Past [substance] user” without any temporal qualifications means that the patient is categorised as a “Past” substance user unless the text states that the patient stopped using the substance less than one year ago.
None	A “None” substance user’s discharge summary indicates that they have never used the substance.
Unknown	A patient’s substance-use status is categorised as “Unknown” if the patient discharge summary does not mention anything about the use of the substance. Indecision between “Current” substance user and “Past” substance user is not included in this category.

Note: Guidelines were adapted from the smoking-challenge annotation guidelines [64]

**Table 2:** Examples of attributes for smoking, alcohol, and drug use.

Substance	Attribute	Example
Smoking	Amount	100 pk, 2 and 1/2 to 3 packs, heavy tob.
	Type	smoking cigarettes, pipe and cigar smoker.
	Frequency	per day, /years.
	Quit-time	stopped smoking in 1994, none since 1967.
	Period	×35 years, for 30 years.
Alcohol	Amount	1–2 drinks, up to one pint.
	Type	two gallons of gin and vodka, glasses of scotch.
	Frequency	one to two times a month, drinks alcohol occasionally
	Quit-time	quit alcohol 25 years ago, last drink 3/2/05.
	Period	for 8 to 9 years, times 33 years.
Drugs	Amount	cocaine abuse.
	Type	current cocaine user, smokes marijuana.
	Frequency	daily, rarely.
	Quit-time	last use seven years ago.
	Period	for three years.

**Table 3:** Annotation summary in terms of number of records for the substance-use statuses of the dataset’s discharge records.

Substance	Record Status			
	Unknown	None	Past	Current
Smoking	939	309	286	205
Alcohol	1111	345	65	218
Drugs	1522	172	17	28

**Table 4:** Substance-use attribute annotation summary in terms of the number of sentences that contain relevant information.

Substance	Sentences	Amount	Type	Frequency	Quit-Time	Period
Smoking	1067	273	72	261	170	69
Alcohol	795	124	43	188	37	7
Drugs	273	29	68	4	6	6

**Table 5:** Past temporal status terms and search methods.

Past Terms	Substance-Use Keyword Distance	Method
Remote	In the same chunk	Chunking
In the past		
In the last		
Previous		
Former	One word after	Window
Prior		
Recovered		
Distant		
Past	Two words before and two words after	Window
Until	All words before	Window
Inactive		
None since		
Discontinued	One word after if not all words before	Window
Quit		
Ago		
Stopped		

**Table 6:** Substance-use status-detection performance using the proposed approach.

Task	Status	Smoking			Alcohol			Drugs		
		Sen	Pre	F1	Sen	Pre	F1	Sen	Pre	F1
Stage 1	Unknown	1	0.9938	0.9969	1	0.9947	0.9974	0.9785	0.9785	0.9785
Stage 2	None	0.9735	0.9910	0.9821	0.9630	0.9848	0.9738	0.9571	0.9853	0.9710
Stage 3	Past	0.9238	0.9700	0.9463	0.7200	0.9000	0.8000	0.7500	1	0.8571
	Current	0.9518	0.9080	0.9294	0.9634	0.9294	0.9461	0.9333	0.7000	0.8000

**Table 7:** Attribute extraction performance of the proposed method.

Attribute	Smoking			Alcohol			Drugs		
	Sen	Pre	F1	Sen	Pre	F1	Sen	Pre	F1
Amount	0.9798	1	0.9898	0.8824	1	0.9375	0.9167	1	0.9565
Type	1	1	1	0.8750	1	0.9333	0.8182	0.9474	0.8780
Frequency	0.9348	1	0.9663	0.9667	1	0.9831	-	-	-
Quit-time	0.9016	0.9483	0.9621	0.7500	0.8571	0.8000	0.7500	0.7500	0.7500
Period	0.9868	0.9782	0.9244	-	-	-	1	1	1

**Table 8:** Substance-use status-detection performance (F1-score) using the examined deep learning approaches and the proposed approach.

Substance	Status	F1-Score Per Method							Train/Test
		Proposed	TE+MLP	TE+BiLSTM	GLV+MLP	GLV+BiLSTM	BERT	BERT (FT)	Samples
Smoking	Unknown	**0.9969**	0.7537	0.6886	0.7358	0.6275	0.0688	0.7611	617/332
	None	**0.9821**	0.2930	0.3322	0.3492	0.3613	0	0.4768	196/105
	Past	**0.9463**	0.2264	0.1176	0.1493	0.2233	0.0921	0.1062	179/97
	Current	**0.9294**	0.1282	0.0682	0.1235	0.0215	0.2072	0.1322	134/73
Alcohol	Unknown	**0.9974**	0.7930	0.7585	0.7909	0.6819	0.5846	0.8000	725/391
	None	**0.9738**	0.3866	0.2682	0.2791	0.3431	0.2793	0.1594	209/113
	Past	**0.8000**	0	0	0.0769	0.0417	0.0263	0	44/24
	Current	**0.9461**	0.1443	0.2455	0.2793	0.1990	0.2020	0.2873	148/79
Drugs	Unknown	**0.9785**	0.9442	0.8971	0.9417	0.3424	0.4271	0.9370	992/535
	None	**0.9710**	0.2424	0.1579	0.1587	0.1970	0.1964	0	105/56
	Past	**0.8571**	0.5000	0.5000	0.5000	0	0	0	11/6
	Current	**0.8000**	0.1818	0.1538	0.1818	0.0444	0	0	18/10

Note: TE: Trainable Embedding, GLV: GloVe Embeddings, FT: Fine-Tuned. In cases where the F1-score for a task is 0, the model did not predict any sample as belonging to that class. Bold denotes the best performance per substance and status.

**Table 9:** Performance comparison in terms of F1-score on the MTSamples dataset.

Substance	Status	Yetisgen et al. [76]	Proposed
Smoking	None	0.9494	**0.9594**
	Past	0.8052	**0.8989**
	Current	0.8043	**0.9043**
Alcohol	None	0.9572	**0.9735**
	Past	0.3529	**0.9043**
	Current	**0.8320**	0.7368
Drugs	None	**0.9761**	0.8333
	Past	-	**0.7500**
	Current	0.7000	**0.8333**

Note: Bold denotes the best performance per substance and status.

**Table 10:** Attribute extraction performance on the MTSamples dataset.

Attribute	Smoking			Alcohol			Drugs		
	Sen	Pre	F1	Sen	Pre	F1	Sen	Pre	F1
Amount	0.9508	1	0.9748	0.8214	1	0.9020	1	1	1
Type	0.9722	1	0.9859	1	1	1	0.7857	1	0.9871
Frequency	0.8727	1	0.9320	0.8438	1	0.9153	-	-	-
Quit-time	0.9000	0.9643	0.931	0.6250	1	0.7692	0.7500	1	0.8571
Period	0.7083	1	0.8293	0.8000	1	0.8889	1	0.9908	0.9954

## Data Availability

The source datasets are publicly available from their respective sources.

## Abbreviations

The following abbreviations are used in this manuscript:

BERT	Bidirectional Encoder Representations from Transformers
Bi-LSTM	Bidirectional Long Short-Term Memory
BRAT	Brat Rapid Annotation Tool
CNN	Convolutional Neural Network
cTAKES	Clinical Text Analysis Knowledge Extraction System
ECOC	Error Correcting Output Codes
EHR	Electronic Health Record
kNN	k-Nearest Neighbours
MedLEE	Medical Language Extraction and Encoding
MLP	Multilayer Perceptron
NHS	National Health Service
NLP	Natural Language Processing
SVM	Support Vector Machines
TF-IDF	Term Frequency–Inverse Document Frequency
UIMA	Unstructured Information Management Architecture
UPMC	University of Pittsburgh Medical Center
